# Oncogenic dysregulation of pre‐mRNA processing by protein kinases: challenges and therapeutic opportunities

**DOI:** 10.1111/febs.16057

**Published:** 2021-06-27

**Authors:** Chiara Naro, Pamela Bielli, Claudio Sette

**Affiliations:** ^1^ Department of Neuroscience Section of Human Anatomy Catholic University of the Sacred Heart Rome Italy; ^2^ Fondazione Policlinico Universitario A. Gemelli IRCCS Rome Italy; ^3^ Department of Biomedicine and Prevention University of Rome Tor Vergata Italy; ^4^ Fondazione Santa Lucia IRCCS Rome Italy

**Keywords:** alternative polyadenylation, alternative splicing, kinase inhibitors, pre‐mRNA‐processing, regulatory phosphorylation

## Abstract

Alternative splicing and polyadenylation represent two major steps in pre‐mRNA‐processing, which ensure proper gene expression and diversification of human transcriptomes. Deregulation of these processes contributes to oncogenic programmes involved in the onset, progression and evolution of human cancers, which often result in the acquisition of resistance to existing therapies. On the other hand, cancer cells frequently increase their transcriptional rate and develop a transcriptional addiction, which imposes a high stress on the pre‐mRNA‐processing machinery and establishes a therapeutically exploitable vulnerability. A prominent role in fine‐tuning pre‐mRNA‐processing mechanisms is played by three main families of protein kinases: serine arginine protein kinase (SRPK), CDC‐like kinase (CLK) and cyclin‐dependent kinase (CDK). These kinases phosphorylate the RNA polymerase, splicing factors and regulatory proteins involved in cleavage and polyadenylation of the nascent transcripts. The activity of SRPKs, CLKs and CDKs can be altered in cancer cells, and their inhibition was shown to exert anticancer effects. In this review, we describe key findings that have been reported on these topics and discuss challenges and opportunities of developing therapeutic approaches targeting splicing factor kinases.

## Graphical abstract

Phosphorylation of RNA polymerase II and regulatory RNA‐binding proteins represents a major modality of pre‐mRNA‐processing regulation. Serine arginine protein kinase (SRPK), CDC‐like kinase (CLK) and cyclin‐dependent kinase (CDK) are the three main families of protein kinases regulating this process. This review reports findings that have highlighted oncogenic alteration of the pre‐mRNA‐processing as a consequence of aberrant regulation of SRPK, CLK and CDK activity and discuss challenges and opportunities of developing therapeutic approaches targeting these kinases.

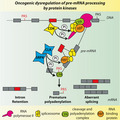

**Abbreviations**
APA: alternative polyadenylation
AS: alternative splicing
CDK: cyclin‐dependent kinase
CLK: CDC‐like kinase
CPSF: cleavage and polyadenylation specific factor
CTD: RNA polymerase II C‐terminal domain
DDR: DNA damage response
PARPi: poly‐adenosyl ribose polymerase inhibitors
PAS: polyadenylation signals
RBPs: RNA‐binding proteins
RNPII: RNA polymerase II
SRPK: serine arginine protein kinase

## Introduction

Most RNAs transcribed in the nucleus serve as vectors of the genetic information encoded in the DNA. The nascent transcripts undergo several steps of co‐ and post‐transcriptional processing that are required for their maturation, including splicing of intronic sequences, 3′ end cleavage and polyadenylation [1, 2]. These processes are mediated by complex macromolecular machineries, namely the spliceosome and the cleavage and polyadenylation complex, whose activity and recruitment to the newly transcribed RNAs are modulated by both transcription dynamics [3, 4] and interaction with hundreds of auxiliary RNA‐binding proteins (RBPs) [5, 6].

Recognition of the exon‐intron boundaries and polyadenylation signals (PAS) are hampered by the degenerate nature of the splice‐site sequences and by the presence of multiple PAS within the transcription unit. For this reason, additional *cis*‐acting sequences, named splicing enhancers and silencers, or proximal and distal sequence elements, respectively, are recognised by specific RBPs that orchestrate the production of multiple transcripts through alternative splicing (AS) and alternative polyadenylation (APA) from most eukaryotic genes [5, 6]. The transcriptome diversity generated by these mechanisms allows fine‐tuning of gene expression under physiological conditions, thus expanding the coding potential and the flexibility of utilisation of the genome. However, errors in these complex processing mechanisms can lead to aberrant expression of RNA isoforms that promote disease onset or progression. Indeed, many human pathologies have been linked to defective pre‐mRNA‐processing events, and dysregulation of these mechanisms are particularly frequent in human cancers [7]. Thus, investigation of the molecular mechanisms underlying the oncogenic alteration of both AS and APA represents a flourishing field of studies which hold the promise to yield novel and more specific anti‐tumoral therapeutic approaches [7, 8]. In this regard, protein kinases regulating pre‐mRNA‐processing represent promising objects of investigation, as they can be potentially targeted by specific enzymatic inhibitors. Notably, several protein kinases have been shown to regulate both RBPs and the RNA polymerase II (RNPII) through phosphorylation, a post‐translational modification that plays a key role in coupling pre‐mRNA‐processing with transcription in response to physiological and pathological signals [9, 10].

Protein phosphorylation affects AS at multiple steps, by modulating the activity of core components of the spliceosome as well as the expression, activity and/or subcellular localisation of regulatory RBPs [9, 11]. The protein kinases that mediate such control include both splicing‐specific kinases, such as the serine arginine protein kinase (SRPK) and CDC‐like kinase (CLK) families, and cell‐signaling kinases, whose activity integrates pre‐mRNA‐processing within cellular responses to specific cues [9]. On the other hand, regulatory phosphorylation of the RNPII is largely mediated by members of the cyclin‐dependent kinase (CDK) family, which target residues of the heptapeptide (YSPTSPS) repeats in its C‐terminal domain (CTD) [12]. Sequential phosphorylation of the CTD by CDKs regulates the kinetics of the transcription cycle (i.e. transition from initiation to elongation, productive elongation and termination). This regulation also affects AS and APA by modulating the window of opportunity between competing splice sites or PAS, respectively [2, 13]. In addition, CTD phosphorylation is also crucial for pre‐mRNA‐processing because it promotes the recruitment of RBPs involved in this mechanism [10]. In line with the extensive links between these processes, overexpression of mutant RNPII with a defective elongation rate, or treatment with CDK inhibitors that block phosphorylation of the CTD, both induced widespread alteration in pre‐mRNA‐processing events [14, 15, 16, 17, 18]. To couple these processes, the CTD acts as a docking platform for the co‐transcriptional recruitment of splicing and cleavage and polyadenylation factors (CPAs) in a phosphorylation‐dependent fashion [10, 19].

In this review, we describe studies that illustrate how SRPKs, CLKs and CDKs contribute to oncogenic dysregulation of pre‐mRNA‐processing, highlighting challenges and opportunities of developing therapeutic approaches targeting their activity.

## The SR‐protein kinase family

SRPK1–3 form a small family of serine‐threonine kinases that specialise in the phosphorylation of serine residues within serine/arginine (S/R) dipeptides enriched in SR proteins, a large family of splicing factors [20, 21].

**Fig. 1:**
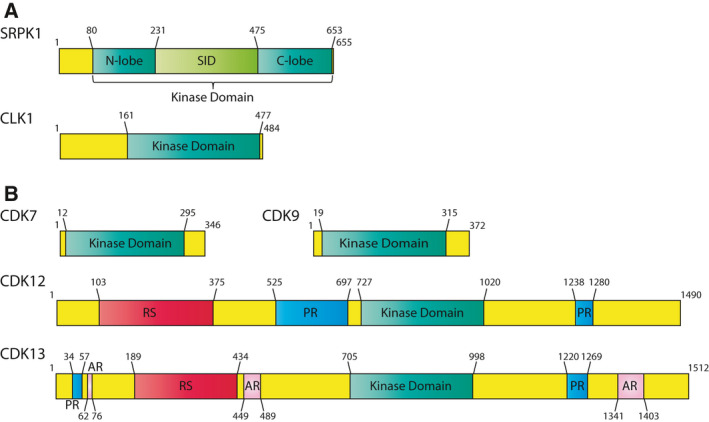
Schematic structures of prototype members of SRPK, CLK and CDK families, implicated in alternative mRNA processing. (A, B) Schematic representation of the functional domains of the splicing factor kinases SRPK1 and CLK1 (A) and of the transcriptional related CDKs, CDK7, CDK9, *CDK12* and CDK13 (B). SRPK1 shows a bi‐lobular kinase domain, interrupted by a unique spacer insert domain (SID), site of interaction with molecular chaperones. CLK1, CDK7 and CDK9 are primarily constituted by their kinase domain. *CDK12* and CDK13 display a RS domain, enriched in Arg/Ser residues and multiple proline‐rich regions (PR). CDK13 also has three alanine rich domains (AR).

**Fig. 2:**
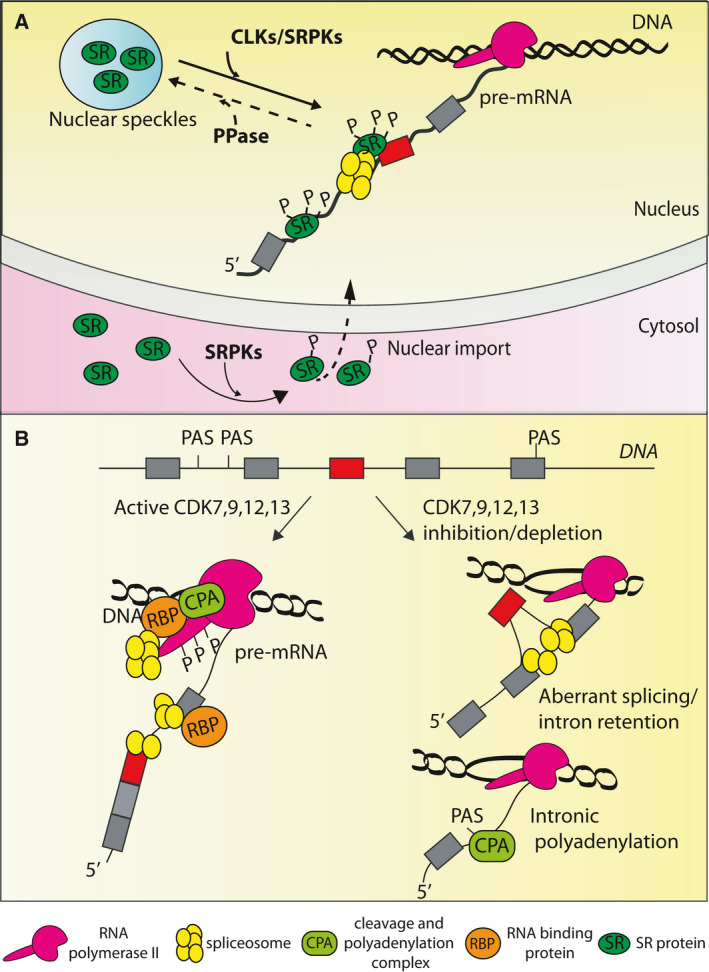
Phosphorylation‐mediated regulation of RNA‐processing. (A) SRPKs and CLKs cooperate in phosphorylation of SR proteins. SRPK‐ and CLK‐activity is counteracted by specific phosphatase (PPase), making SR protein‐phosphorylation reversible. This modification modulates SR proteins shuttling between nuclear speckles and active site of pre‐mRNA‐transcription and processing. It also affects SR protein physical and functional interaction with the spliceosome. In the cytoplasm, SRPKs‐mediated phosphorylation regulates SR protein nuclear import. (B) Schematic representation of a eukaryotic gene, displaying multiple constitutive (grey boxes) and alternative exons (red boxes) and several PAS. Recognition of these elements during pre‐mRNA‐processing is modulated by transcriptional CDKs (CDK7/9/12/13). These kinases phosphorylate the RNPII CTD at different residues and with different timing. These modifications affect transcription dynamics and RNPII interactions with RNAprocessing factors (i.e RBP, spliceosomal proteins and CPA). Inhibition or depletion of CDK7/9/12/13 alter co‐transcriptional RNA‐processing, favouring aberrant AS, intron retention and polyadenylation at premature PAS.

SRPK1 is the prototype and the most investigated member of this family (Fig. 1A). Its structure is characterised by a bipartite kinase domain, separated by a unique spacer insert domain (SID) [20, 21]. In non‐stimulated cells, SRPK1 is mainly localised in the cytoplasm, where it is held by a strong cytosolic retention signal within the SID and by the interaction with molecular chaperones [22, 23, 24]. In the cytoplasm, SRPK1 phosphorylates SR proteins and stimulates their nuclear import by increasing their affinity for their specific transportin SR2 [25] (Fig. 2A). Different intra‐ and extracellular signals lead to dissociation of chaperones from SRPK1 and induce its nuclear translocation [22, 24]. In the nucleus, SRPKs cooperate with CLKs to regulate shuttling of SR proteins between nuclear speckles and nucleoplasm by phosphorylation of different S/R residues [26, 27] (Fig. 2A). Moreover, direct interaction with CLK1 was found to stabilise the nuclear localisation of SRPK1 and to promote the release of SR proteins from CLK1 upon their phosphorylation, thus allowing timely association with spliceosomal components [28, 29, 30]. In addition to SR‐proteins, SRPKs also regulate the activity of other splicing factors, such as TRA2B [31] and RBM4 [32, 33]. Moreover, a systematic study combining *in vitro* kinase assays with affinity purification‐mass spectrometry revealed that other RNA‐processing factors, including hnRNPs, spliceosomal proteins and components of the exon junction complex (EJC), are substrates of SRPKs [34]. In particular, both SRPK1 and SRPK2 were found to phosphorylate RBM8A (or Y14) [34], an EJC component whose phosphorylation regulates its interaction with other EJC proteins and with mRNA decay factors [35, 36]. Lastly, the cleavage and polyadenylation‐specific factors CPSF6 and CPSF7 (also known as CFIm68 and 59) are also phosphorylated by SRPKs. This post‐translational modification might affect the polyadenylation process, as suggested by the widespread changes in APA observed in cells expressing an hypo‐phosphorylated CPSF6[S8YA] mutant [37, 38]. Thus, although a comprehensive analysis of post‐transcriptional processes other than splicing is still lacking, these observations suggest that SRPKs regulate and coordinate various stages of pre‐mRNA processing.

**Fig. 3:**
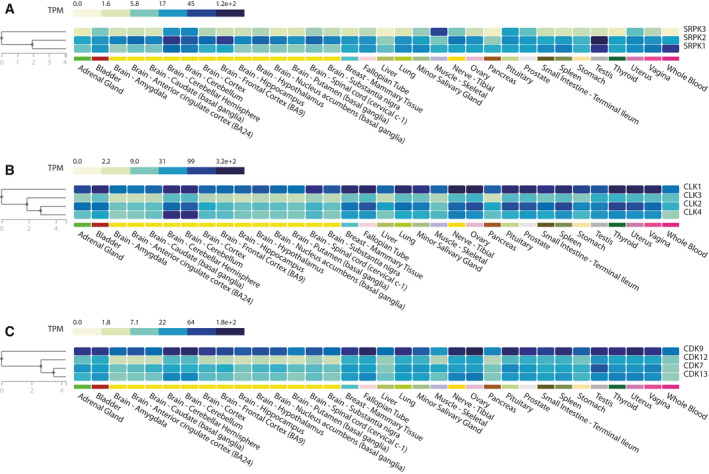
SRPK‐, CLK‐ and transcriptional CDK‐expression levels in human tissues. (A–C) Clustergrams illustrating expression levels of indicated members of the SRPK (A), CLK (B) and CDK (C) families, in human tissues. Data and figures were retrieved from the Genotype‐Tissue Expression (GTEx) Portal. Gene expression is shown in Transcripts Per Million (TPM). The GTEx Project was supported by the Common Fund of the Office of the Director of the National Institutes of According Health, and by NCI, NHGRI, NHLBI, NIDA, NIMH, and NINDS. The data used for the analyses described in this manuscript were obtained from: the GTEx Portal on 04/30/21.

SRPK1 and 2 are ubiquitously expressed in human tissues, with the highest expression levels in the brain and testes, whereas SRPK3 is primarily expressed in brain and muscle [39, 40] (Fig. 3A). In line with this pattern of expression, *Srpk1* genetic ablation is an embryonic lethality in the mouse, whereas knockout of *Sprk3* elicits a type‐2 specific myopathy in murine models [40, 41]. To date, no direct information is available on the impact of SRPKs on developmentally‐regulated splicing programmes. However, the peculiar higher expression of both SRPK1 and 2 in brain and testes (Fig. 3A) suggests their implication in the generation of the large transcriptome diversity in these organs [42]. Remarkably, SRPK1‐mediated phosphorylation R/S dipeptides of protamine 1 is crucial to ensure the protamine‐to‐histone exchange underlying reactivation of the paternal genome after fertilisation [43]. Moreover, SRPK1‐mediated phosphorylation of the ubiquitin ligase RNF12 was shown to regulate a neurodevelopmental‐specific transcriptional programme [44]. Collectively, these observations highlight the multifunctional role played by SRPKs in the regulation of developmental processes in higher eukaryotes.

## The role of SRPKs in human cancers

SRPK1‐upregulation was documented in several human solid tumours [45, 46, 47, 48] and in leukaemia [49, 50]. In most of these cancers, SRPK1‐upregulation was correlated with poor outcome and negative prognostic factors, such as higher tumour grade and stage or metastatic behaviour [46, 47, 51, 52]. However, exception to this trend was reported in patients affected by testicular germ cell tumours and paediatric retinoblastoma, for whom high expression of SRPK1 apparently correlates with better prognosis and increased sensitivity to chemotherapy [53, 54]. Such opposite trends could reflect changes in post‐translational modifications of SRPK1, which modulate its activity. For instance, phosphorylation by casein kinase 2 (CK2) o LIMK2 was shown to enhance SRPK1 activity [55, 56], whereas TIP60‐mediated acetylation inhibits SRPK1 auto‐phosphorylation and reduces its stability [57]. Thus, it is possible that post‐translational modifications occur differently in cancers showing opposite prognostic potential of SRPK1, and contribute to its functional impact. In this regard, it is interesting to mention that both knock‐out and overexpression of SRPK1 were reported to affect oncogenic features of mouse embryonic fibroblasts (MEFs). Ablation of SRPK1 promoted neoplastic transformation of MEFs, whereas its overexpression enhanced anchorage‐independent cell growth [41]. Notably, hyper‐activation of the AKT pathway was suggested to underlie this ambivalent behaviour. SRPK1 interacts with the AKT kinase and its regulatory phosphatase PHLPP1, and both high and low SRPK1 levels were shown to interfere with a negative feedback mechanism that normally tunes the AKT pathway [41]. These observations suggest that splicing‐independent regulatory activities of SRPK1 might also concur with its multifaceted behaviour observed in human cancers.

The levels of SRPK1 in cancer cells could be controlled at the transcriptional level by the Wilms' tumour suppressor (WT1), a zinc‐finger transcription factor shown to either repress or activate SRPK1 expression in different cellular contexts. Indeed, WT1 repressed SRPK1 transcription in a non‐tumoral Denys Drash Syndrome podocyte cell line [58], whereas a more recent study reported that WT1 acts as a transcriptional activator of SRPK1 in prostate cancer and leukaemia cells [59]. This positive function is inhibited by the interaction of WT1 with the transcriptional co‐repressor BASP1 [60], highlighting the relevance of the cellular context for the regulation of SRPK1 transcription. In addition, SRPK1 expression is repressed at post‐transcriptional level by several microRNAs, such as mir‐9 [61], mir‐99 [62], mir‐1296 [63], mir‐216b [64], mir‐126 [65] and miR‐485‐p [66]. Changes in expression of these microRNAs might contribute to modulate the cellular levels of SRPK1 in different cancer types. For instance, it was proposed that activation of the transcription factor HIF1α upon hypoxic conditions causes the downregulation of mir‐1296 and miR‐485‐p [63, 66]. Hence, transcriptional regulation of specific microRNAs by HIF1α might induce the upregulation of SRPK1 under hypoxia that has been documented in multiple cancer cells [67, 68].

SRPK1 expression and/or activity was shown to promote several of the hallmarks of cancer, including proliferation, resistance to apoptosis, migration and angiogenesis [46, 47, 69, 70, 71, 72, 73]. In most cases, the pro‐tumoral activity of SRPK1 was associated with modulation of select splice‐variants in cancer cells [46, 47, 69, 70, 71, 72, 73]. For instance, depletion of SRPK1 promoted the splicing of a truncated isoform of the *MAP2K2* gene and of a shorter isoform of the apoptotic regulator *MCL‐1*, thus enhancing apoptosis and susceptibility to chemotherapeutic agents [33, 46]. One of the most investigated targets of SRPK1 is the vascular endothelial growth factor (*VEGF*) gene. Alternative usage of a proximal or distal splice site in *VEGF* exon 8 regulates the balance between pro‐ (VEGF165) and anti‐angiogenic (VEGF165b) splice variants [72]. Upregulation of SRPK1 drives phosphorylation and nuclear translocation of the splicing factor SRSF1, which enhances splicing of the pro‐angiogenic VEGF165 isoform in Wilms Tumour [58], melanoma [73] and prostate cancer [47]. More generally, transcriptome profiling approaches have revealed a widespread impact of SRPK1 activity on splicing regulation [50]. Genetic or pharmacologic inhibition of SRPK1 affects more than 2000 splicing‐regulated genes in the leukaemic cell line THP1 [50]. Among them, SRPK1 inhibition caused a cancer‐relevant switch in the pre‐mRNA of the transcriptional regulator *BRD4*. The long BRD4 isoform displayed reduced chromatin‐binding and was proposed a potential therapeutic vulnerability of leukaemia cells. Indeed, SRPK1 inhibition synergised with inhibitors of BRD proteins in reducing the growth of leukaemia cells [50]. This finding strongly suggests that characterisation of the transcriptome signature regulated by SRPK1 in other tumour types could provide valuable insights regarding its oncogenic activity, as well as suggestions about potential targets to be tested in combined therapeutic approaches.

## The CDC‐like kinase family

The CLK family comprises four members (CLK1–4) of SR protein kinases, which are ubiquitously expressed in human tissues (Fig. 3B). They are characterised by a C‐terminal kinase domain and an N‐terminal disordered domain, which mediates protein‐protein interactions (Fig. 1A). CLKs differ from SRPKs for their preferential localisation in the nucleus and for their broader spectrum of activity. Indeed, these kinases also phosphorylate S‐K or S‐P dipeptides, in addition to S‐R dipeptides [27, 74, 75]. As previously mentioned, by virtue of these differences in localisation and specificity, CLKs cooperate with SRPKs in phosphorylating and regulating the activity of SR proteins [26, 27, 28, 29, 30] (Fig. 2A). In particular, CLK‐mediated phosphorylation enhances the release of SR proteins from nuclear speckles and promotes their interaction with the U1 snRNP [28, 76], thus increasing their splicing activity. Notably, the activity of CLK1 and CLK4 is highly susceptible to physiological temperature changes. This regulation relies on the intrinsic structural features of CLKs, which allow reversible rearrangements in their kinase activation segment [77]. By virtue of this regulation, CLK1 and CLK4 were recently shown to act as temperature sensors, which couple pathological and circadian oscillations of the body temperature with global regulation of gene expression and splicing by regulating SR‐protein phosphorylation [77, 78]. CLK‐mediated phosphorylation of SR proteins is also crucial for the regulation of splicing of the so‐called ‘detained’ introns in murine embryonic stem (ES) cells [79]. Detained introns are defined as a class of evolutionary conserved introns which are retained in highly stable, nuclear polyadenylated transcripts [79]. Post‐transcriptional splicing of detained introns is activated upon specific cellular cues and positively regulates the expression of parent genes [79].

CLKs phosphorylate also other splicing factors, such as TRA2B [80, 81] and SPF45 [82]. For instance, CLK2‐mediated phosphorylation was shown to modulate the feedback mechanism by which TRA2B auto‐regulates its own splicing and expression levels [80]. Although CLKs, and in particular CLK1, are prevalently localised in the nucleus [28, 83], these kinases can also function in the cytoplasm [84]. Among the cytoplasmic targets, CLKs can phosphorylate the tyrosine phosphatase PTP1B [85] and the junctional proteins PKP2, PPHLN1 and PNN [34] *in vitro*. Moreover, the mitotic kinase Aurora B and B56β, a regulatory subunit of the PP2A complex, were also reported to be functionally relevant substrates of CLKs in live cells [86, 87]. Phosphorylation of Aurora B by CLK1, 2 and 4 was shown to be essential for proper cytokinesis [86], while CLK2‐mediated phosphorylation of B56β promoted assembly of the functional PP2A complex by regulating an inhibitory feedback loop impinging on the AKT kinase [87].

## The role of CLKs in human cancers

Upregulation of CLK1–4 expression levels was reported in multiple cancer types, including breast [88], colon, renal, and lung carcinomas [89]. Moreover, high expression levels of CLK1 or CLK2 were associated with negative prognosis in renal carcinoma and glioblastoma, respectively [89, 90]. The increased expression of CLK2 in breast cancer was correlated with the amplification of its locus [88], whereas the cause of CLK1 upregulation in tumours has not been investigated yet. However, CLK1 protein expression is regulated by a ubiquitin‐mediated degradation mechanism during cell cycle progression, resulting in a peak of expression during the G2/M phase [89]. Thus, the increased mitotic index of tumours with respect to normal tissue, combined with the higher CLK1 transcript levels, may account for the observed upregulation of this kinase in human cancers. Furthermore, CLK1 and CLK4 expression levels are also regulated by post‐transcriptional splicing of the introns flanking their exon 4. These introns are retained in a large fraction of polyadenylated transcripts in mouse cells, and their splicing is activated upon cellular stresses, such as heat or osmotic shock [91]. CLK1/4 appear to control their own pre‐mRNA, as their chemical inhibition promoted splicing of the retained introns [79, 91]. CLK1 also auto‐regulates AS of its own exon 4, of which the skipping generates a truncated and catalytically inactive protein isoform [92]. Notably, *CLK1* intron 4 is variably retained and exon 4 largely excluded, also in a subset of human cancer cell lines and this regulation is reverted by stress conditions [93]. Thus, alteration of this conserved auto‐regulatory mechanism might also contribute to changes in CLK1 expression and activity in cancers.

Downregulation or chemical inhibition of CLKs impairs growth and metastatic properties of tumours and these effects were correlated with the modulation of specific splice variants [88, 90, 94, 95, 96]. For instance, CLK inhibition in a glioblastoma model favours splicing of a cytoplasmic isoform of the oestrogen‐related receptor β (*ERR‐B*) gene, named ERR‐ β 2, which in turn inhibits cancer cell growth and migration [95]. Moreover, two distinct CLK inhibitors were reported to increase the skipping of exon 7 in the *RPS6KB1* gene, a splicing event directly correlated with the anti‐proliferative effects of these drugs in breast cancer models [94, 96]. At genome‐wide level, CLK inhibitors modulated a subset of AS events that are dysregulated across tumour types displaying CLK1 upregulation [89], suggesting their efficacy in reverting CLK‐mediated oncogenic features. Interestingly, ˜ 65% of the CLK‐dependent AS events were also susceptible to periodic variation across the cell‐cycle. Thus, the splicing activity of CLKs may contribute to determine the proper timing of cell‐cycle progression [89]. This observation, together with the cell cycle‐dependent expression of CLKs and their role in Aurora B phosphorylation [86, 89], suggests that CLK inhibitors might represent promising therapeutic options for cancers treated with drugs that interfere with mitosis, such as taxanes for triple‐negative breast cancers [97].

Transcriptomic analyses in different cancer cell lines revealed that chemical inhibition of CLKs results in pervasive intron‐retention patterns [89, 98]. Interestingly, inefficient splicing of detained introns represents a vulnerability for glioblastoma cells. It was shown that inhibition of PRMT5, an arginine methyl transferase that methylates spliceosomal proteins and is required for spliceosome maturation [99, 100] resulted in an accumulation of detained introns and elicited strong anti‐tumoral effects [101]. These observations suggest that CLK inhibitors might synergise with other spliceosome inhibitors in halting the growth of tumours that, similarly to glioblastoma, display high levels of detained introns [102]. In this view, it will be interesting to evaluate whether MYC‐driven tumours, which are known to be particularly susceptible to spliceosome inhibition [103], are also particularly sensitive to the anti‐tumoral activity of CLK inhibitors and whether these drugs synergise in combined regimens. In support of this hypothesis, a novel potent ATP‐competitive inhibitor of CLK2 (T‐025; N2‐methyl‐N4‐[pyrimidin‐2‐ylmethyl]‐5‐[quinolin‐6‐yl]‐7H‐pyrrolo[2,3‐d]pyrimidine‐2,4‐diamine) exhibited particular efficacy in MYC amplified cancer cell lines [94]. However, in this initial study T‐025 was not found to induce massive intron‐retention as observed by other spliceosome inhibitors [94]. Thus, further investigation of the mechanism(s) underlying this susceptibility are still necessary to elucidate its full potential [104]. Another interesting observation is that a new generation ATP‐competitive inhibitor of CLKs, named T3 [4‐(2‐methyl‐1‐(4‐methylpiperazin‐1‐yl)‐1‐oxopropan‐2‐yl)‐N‐(6‐(pyridin‐4‐yl)imidazo[1,2‐a]pyridin‐2‐yl)] promotes the formation of conjoined gene transcripts, as a consequence of aberrant RNA‐processing between transcripts of distinct genes [98]. This pattern was correlated to the enrichment of specific RNA motifs in the last and second exons of upstream and downstream partners, respectively. These motifs were predicted to be recognised by RBPs that are known to affect both AS and APA processes [98], such as U2AF2 and KHDRBS1 [105, 106]. Moreover, mass‐spectrometry assays documented a direct interaction between CLK2 and these RBPs as well as with core components of the cleavage and polyadenylation complex, including CPSF7 [98]. Since the alternative last exon was the second most abundant AS pattern regulated by T‐025 after the cassette exon [94], these observations hint at a possible involvement of CLKs also in 3′‐end RNA‐processing regulation and suggest that impairment of this function might concur with the anti‐tumoral effects elicited by their chemical inhibition.

## The cyclin‐dependent kinase family

Cyclin‐dependent kinases are a family of serine‐threonine kinases, requiring association with regulatory cyclins to exert their activity. The human genome comprises 21 CDKs and five CDK‐like (CDKL) genes, which can be classified in cell‐cycle‐related and transcriptional‐related subfamilies on the basis of their substrate specificity [107]. In particular, the transcriptional CDKs ensure orderly progression of the sequential phases of the transcription cycle, as well as its efficient and accurate coordination with the pre‐mRNA‐processing events that are required for proper gene expression (Fig. 2B) [108, 109, 110]. Among them, *CDK7*, *CDK9*, *CDK12* and *CDK13* (Fig. 1B) are the kinases whose regulatory impact on pre‐mRNA‐processing has been more extensively investigated. All these CDKs are ubiquitously expressed in human tissues (Fig. 3C) and act on similar, yet not overlapping, steps of transcription and pre‐mRNA‐processing.

### CDK7

CDK7 function is essential *in vivo* and its ablation leads to defects in cell cycle progression and early embryonic lethality [111]. Moreover, conditional knockout of *CDK7* in adult mice indicated that this kinase is essential for the homeostasis of tissues with elevated cellular turnover, which undergo exhaustion of self‐renewal capacity and premature ageing in its absence, whereas CDK7 appears to be dispensable in tissues characterised by low proliferation [111]. CDK7 associates with MAT1 and Cyclin H in the cytoplasm as part of the CDK‐activating kinase (CAK) complex, and regulates phosphorylation of cell‐cycle CDKs [111, 112]. In line with this role, loss of CDK7 was reported to induce mitotic defects in lower organisms, such as yeast and *Caenorhabditis elegans* [113, 114]. Moreover, selective inhibition of an analog‐sensitive CDK7 allele (CDK7*as*) in human colon cancer cells impaired activation of CDK1 and CDK2 and cell cycle progression [115]. On the other hand, in the nucleus the CAK complex is tethered to the general transcription factor TFIIH and facilitates RNPII escape from the preinitiation complex (PIC) in promoter regions. This latter function is operated through phosphorylation of the RNPII CTD at Ser5 and of CDK9, the catalytic subunit of the transcription elongation factor P‐TEFb [116, 117]. More recently, CDK7 was also reported to phosphorylate several splicing factors, including the U2 snRNP protein SF3B1 and the general splicing factor U2AF2 [118]. In addition, CDK7 also phosphorylates *CDK12* and CDK13 [118], which are involved in the phosphorylation of the RNPII CTD at Ser2 within the gene body and play a role in RNA processing regulation (see below). These findings, together with the observation of a significant overlap between AS changes elicited by CDK7 inhibition and the SF3B1 inhibitor Pladienolide B [118], provide strong evidence for a direct and/or indirect role for CDK7 in splicing regulation.

### CDK9

CDK9 also mediates the phosphorylation of the RNPII CTD at Ser2 [108]. Loss of CDK9 expression *in vivo* causes embryonic lethality [119] and its activity was shown to contribute to developmental and tissue‐specific differentiation programme types, such as myogenic and lymphoid differentiation [120, 121]. Together with its partner Cyclin T1, CDK9 is a key component of the P‐TEFb complex involved in transcription elongation. Upon promoter clearance, association with the DRB sensitivity‐inducing factor DSIF (a heterodimer composed of the Spt5 and Spt4 subunits) and negative elongation factor NELF causes the stalling of the elongating RNPII ˜ 50–100 nt downstream of the transcription start site. At this stage, CDK9‐mediated phosphorylation of Spt5, NELF and the CTD triggers the release of the RNPII from promoter‐proximal pausing, allows its transition into the gene body and promotes transcript elongation [122, 123, 124]. Notably, chemical inhibition of CDK9 also interferes with co‐transcriptional splicing and with the 3′‐end RNA cleavage and polyadenylation [125] (Fig. 2B). This defect is likely a consequence of the altered recruitment of regulatory factors, such as U2AF2 [19] and the CPAs PCF11 [19], CSTF2 (also known as CSTF64) and SSU72 [126]. CDK9 also directly phosphorylates and enhances the activity of XRN2, the 5′–3′ exonuclease that degrades the 5′ uncapped RNA generated downstream of the cleavage site during 3′‐end pre‐mRNA‐processing [127]. Depletion or inhibition of CDK9 impaired the localisation of XRN2 on the chromatin and increased the read‐through transcription consistently with inefficient transcription termination [127]. These observations indicate that timely phosphorylation by CDK9 of different targets is essential for the coordination of transcription and processing of the nascent transcripts.

### *CDK12*/CDK13

*CDK12* and CDK13 are highly homologous kinases characterised by their larger size with respect to the other transcriptional CDKs (Fig. 1B). *CDK12* and CDK13 associate with Cyclin K and play a partially redundant role in the regulation of transcription elongation [128]. These CDKs, as well as their partner Cyclin K, are highly expressed in murine ES cells and their levels decrease when differentiation begins [129]. Notably, knockdown of *CDK12* or CDK13 reduced the expression of transcription factors involved in stemness, such as OCT4 and SOX2, indicating their role in self‐renewal of stem cells [129]. Furthermore, loss of *CDK12* function impaired expression of DNA damage response (DDR) genes, thus promoting genomic instability and apoptosis in both ES and neural progenitor cells [130, 131]. Collectively, these observations highlight the critical role played by *CDK12* and CDK13 in embryogenesis [129, 130, 132] and neurogenesis [131].

*CDK12* and CDK13 phosphorylate the RNPII CTD at Ser2 in the gene body and towards the 3′‐end of the transcription units [109, 110, 133]. Within proximity of the PAS, the local increase in RNPII Ser2 phosphorylation induced by *CDK12* promotes the recruitment and stable association of the CPAs CSTF3 (also known as CSTF77) and CPSF3 (also known as CPSF73), thus enhancing execution of 3′‐end processing [134, 135]. *CDK12* activity is also involved in suppression of intronic premature polyadenylation (IPA) at cryptic PAS [136, 137] (Fig. 2B). This function likely involves both its activity as regulator of transcription elongation and direct phosphorylation of RNA‐processing factors, including the CPAs CPSF7 and CSTF2 and the U1snRNP component *SNRNP70* [137]. *CDK12* inhibition was reported to particularly affect long genes displaying a lower ratio of U1snRNP‐binding sites near intronic PAS [137]. This observation may suggest that the functional interaction between *CDK12* and *SNRNP70* potentiates the ability of U1snRNP to recognise and repress cryptic intronic PAS, thus avoiding premature termination of transcription [138]. Importantly, genes involved in the DDR were enriched among the transcripts undergoing premature cleavage and polyadenylation in *CDK12* null cells [136, 137]. Together with the overall reduction in expression of DDR genes [130], this finding explains the increased sensitivity of *CDK12* null cells to DNA damaging drugs, such as poly‐adenosyl ribose polymerase inhibitors (PARPi) [139, 140]. This observation is clinically relevant, as PARPi are usually applied only to cancer patients bearing mutations in *BRCA1/2* or other DDR genes. In this scenario, it is conceivable that combined treatment with *CDK12* inhibitors may render susceptible to PARPi also cancer cells that are proficient for the DDR pathway (see below). In addition to SNRNP70, *CDK12* was shown to interact with several other splicing factors [128, 137, 141, 142, 143]. This peculiarity of *CDK12*, as well as of CDK13, with respect to other CDKs may relate to the presence in their structure of an RS domain (Fig. 1B), which serves as a protein‐protein interaction domain in many splicing factors. Moreover, both *CDK12* and *CDK13* were shown to localise in nuclear speckles [144, 145], which are sub‐nuclear membrane‐less organelles where several SR proteins and splicing factors accumulate. Thus, *CDK12* and *CDK13* are particularly suited for efficiently coupling transcription and pre‐mRNA‐processing events. In support of this notion, depletion or chemical inactivation of these CDKs caused widespread effects on AS regulation in cancer cells, with a particular impact on the selection of alternative last exons (ALE) in long genes characterised by a large number of exons [128, 141].

## The role of transcriptional CDKs in human cancers

Given the key role played by the transcriptional CDKs in the regulation of gene expression at multiple layers, their role in human cancers has been extensively investigated. Overexpression of *CDK7* was documented in several cancers and it was shown to predict poor prognosis and reduced survival [146, 147, 148, 149, 150, 151]. In pancreatic cancer, *CDK7* activity is particularly important for cancer cells in which MYC is upregulated, possibly due to the general increase in transcription driven by this oncogenic transcription factor [152]. Notably, MYC‐driven cancers were shown to be more susceptible to splicing inhibition [103], suggesting that the effects of *CDK7* on splicing regulation may contribute to its oncogenic role in cancers that rely on MYC.

An interesting example of the multiple impacts that CDKs can exert on tumorigenesis is provided by *CDK12*, for which both amplification and deletion have been linked to tumorigenesis. In breast cancer cells, *CDK12* is frequently amplified together with its neighbouring *HER2* gene and it was shown to drive tumour initiation and progression by activating signaling pathways that promote self‐renewal of cancer stem cells [153]. Furthermore, inhibition of *CDK12* activity was shown to enhance the anticancer efficacy of different HER2‐targeting treatments in HER2^+^ breast cancer cells [153, 154]. By contrast, deletion and/or null mutations in *CDK12* were reported in ˜ 5% of prostate and ovarian cancer patients, where this genetic alteration is correlated with worse prognosis, thus indicating a putative tumour suppressor role for *CDK12* in these cancers [155]. Notably, inactivation of *CDK12* is associated with a distinct phenotype of the tumour, characterised by elevated genomic instability, tandem duplications and possible immunogenicity [156, 157]. The elevated genomic instability is likely due to repression of the DDR pathway in cells lacking *CDK12* activity, which impairs repair of lesions and allows accumulation of genomic aberrations. Thus, the differential outcome of *CDK12*‐inactivation reported in different cancers may actually rely on the same activity of this kinase. Short‐term inactivation of *CDK12* – an essential gene in mammals – is deleterious for cancer cells and exposes them to vulnerability towards several anticancer agents, particularly those impinging on the DDR [136, 137, 140]. However, in the presence of stable knockout of *CDK12* activity, it is likely that a few surviving cancer cells accumulate large genomic aberrations that may ultimately create new oncogenes and generate a highly resistant phenotype, as observed in advanced prostate cancer. In support of this hypothesis, tandem genomic duplications in metastatic prostate cancers were found in regions harbouring an intergenic enhancer element upstream of the androgen receptor gene (*AR*) and near the *MYC* gene, both loci‐encoding transcription factors that play a crucial oncogenic role in prostate cancer and whose overexpression has been associated with dysregulated transcription and RNA‐processing [158, 159].

Collectively, these observations indicate that CDKs coordinate mRNA‐transcription and processing through modulation of transcription dynamics, which affects the window of opportunity for the recruitment of pre‐mRNA‐processing factors, as well as through the direct phosphorylation and modulation of the activity of such factors. Inhibition of these CDKs may be particularly important for tumours relying on altered transcriptional regulation, such as those driven by oncogenic transcription factors like breast (MYC) and prostate (AR) cancers and Ewing sarcomas (EWS‐FLI1).

## Inhibition of splicing factor kinases: emerging therapeutic opportunities

Dysregulation of RNA‐processing is an emerging hallmark of cancer and represents an element of vulnerability that could be exploitable therapeutically [160]. In support of this notion, the spliceosomal SF3B complex was identified as the target of well‐known anticancer agents such as pladienolides, sudemycins and meayamycins [161]. In parallel, due to their druggable enzymatic nature, large efforts have also been devoted to the development of molecules that specifically inhibit splicing factor kinases. The efficacy of SRPK, CLK and CDK inhibitors has now been tested in multiple preclinical studies and hold promise that these drugs can represent valuable anti‐neoplastic agents, especially for cancer types that are driven by transcriptional and co‐transcriptional dysregulation of gene expression programmes [162]. Herein, we will briefly describe the current studies that are attempting to translate the inhibitors of these splicing factor kinases into the clinic (Table 1).

**Table 1:** SRPKs, CLKs and CDKs as inhibitors in clinical trials. Data reported in this table were obtained from https://clinicaltrials.gov/ct2/home.

Kinase family	Inhibitor	Gene target	Indications	Phase	Clinical trial
SRPK	SCO‐101	SRPK1	Metastatic colorectal cancer	I, II	NCT04247256
			Pancreatic ductal adenocarcinoma	I, II	NCT04652206
CLK	SM08502	CLK1–4	Advanced solid tumours	I	NCT03355066
CDK	AZD4573	CDK9	Advanced haematological malignancies	I	NCT03263637
			Advanced haematological malignancies	I, II	NCT04630756
	KB‐0742	CDK9	Solid tumours, Non‐Hodgkin Lymphoma	I	NCT04718675
	SY‐1365	CDK7	Advanced solid tumours, ovarian cancer, breast cancer	I	NCT03134638
	SY‐5609	CDK7	Advanced solid tumours, breast cancer, small‐cell lung cancer	I	NCT04247126
	CT7001	CDK7	Advanced solid malignancies	I, II	NCT03363893
	THZ531	*CDK12* and 13	Ovarian cancer organoids	observational	NCT04555473

### SRPKs

Pharmacological inhibition of SRPK1 activity was shown to exert anti‐angiogenic effects, suggesting the potential value of this approach for anti‐cancer therapies. The ATP‐competitive inhibitor SRPIN430 displays high selectivity for SRPK1 and SRPK2 [163], reduces SR protein phosphorylation and inhibits SRSF1‐dependent splicing of the pro‐angiogenic VEGF variant in cultured cell lines [58]. In line with these observations, SRPIN430 treatment *in vivo* reduces growth of melanoma xenografts, an effect that was accompanied by reduced expression of VEGF and impaired micro‐vascularisation [73]. Moreover, this inhibitor reduced metastasis‐related cellular traits in triple‐negative breast cancer cell lines [56], thus recapitulating the effects observed with stable silencing of SRPK1 [51]. In this latter study, however, the anti‐tumoral effects of SRPK1 depletion was correlated with impairment of the NF‐κB signaling pathway, whereas no significant splicing changes were identified [51]. Thus, SRPK inhibitors might be able to interfere also with splicing‐unrelated activities of SRPK1. Two other ATP competitive inhibitors of SRPK1, SPHINX and its derivative SPHINX‐31, elicited anti‐tumoral effects in models of leukaemia and solid tumours [50, 164, 165]. Importantly, SPHINX‐31 did not affect normal haematopoiesis *in vivo* [50], thus providing encouraging results in relation to its clinical safety. SCO‐101 is a drug described as an inhibitor of ATP‐Binding Cassette (ABC) efflux pumps and of SRPK1 [166], which displayed anti‐cancer potential in combination with docetaxel in triple negative breast cancer cells [167]. More recently, an irreversible SRPK1 inhibitor (SRPKIN) was reported to cause strong antiangiogenic effects in a mouse model of choroidal neovascularisation, a form of age‐related macular degeneration that is correlated to potent VEGF‐splicing modulation [168]. This finding suggests that SRPKIN could display anti‐tumoral activity and may pave the ground for future investigation of this drug in the oncological setting.

Two clinical trials testing the efficacy of an SRPK1 inhibitor in human cancer are currently reported as actively recruiting. In both trials, the drug under evaluation is SCO‐101 and the primary outcome is the evaluation of safety and toxicity of the combination with current chemotherapeutic regimens. The first study (NCT04247256; Table 1) combines SCO‐101 treatment with FOLFIRI (folinic acid, 5‐fluorouracil and irinotecan) in patients affected by metastatic colorectal cancer. Patients recruited for this trial have acquired resistance to FOLFIRI treatment, possibly due to the increased efflux of drugs by cancer cells. The second trial (NCT04652206; Table 1) is an open‐label dose‐escalating phase Ib study of SCO‐101 in combination with gemcitabine and nab‐paclitaxel. This study recruits patients diagnosed with locally‐advanced pancreatic ductal adenocarcinoma (PDAC) who are ineligible for surgery. The rationale of both trials is based on the ability of SCO‐101 to inhibit ABC transporters and possibly revert drug efflux, thus enhancing the efficacy of chemotherapy. However, the predicted inhibition of SRPK1 and of splicing regulation is also thought to contribute to the anti‐cancer efficacy of these treatments. This is particularly appealing in PDAC, where upregulation of the SR protein SRSF1 was shown to confer increased resistance to gemcitabine [169].

### CLKs

Several CLK inhibitors have been developed and tested for their anti‐tumoral activity to date (see [170] for an extended review). A major clinical interest resides in orally available CLK inhibitors, such as the aforementioned T‐025 [94] or the newly developed SM08502 [171]. The latter is a potent CLK2/3 inhibitor displaying striking inhibitory effects on the growth of colorectal cancer cell lines and xenograft models [171]. The anti‐cancer effects of SM08502 were associated with inhibition of SR protein phosphorylation and promotion of aberrant splicing of key regulatory genes belonging to the WNT pathway [171]. SM08502 is currently being investigated in a Phase 1, open‐label dose‐escalation study aimed at evaluating its safety, tolerability, pharmacokinetics and pharmacodynamics in patients with advanced solid tumours (NCT03355066; Table 1). Results from this study will provide insightful information about the potential application of SM08502 in cancer therapy and may pave the ground for testing additional CLK inhibitors. For instance, TG693 is an orally available CLK1 inhibitor that was reported to restore proper splicing of the dystrophin gene in an immortalised cell line from a Duchenne dystrophy patient [172]. Nevertheless, its activity as an anti‐tumoral agent has not been evaluated to date. Given the key role played by CLKs in splicing regulation, it is likely that these inhibitors will prove to be particularly potent if proper selection of patients will take into account hallmarks of vulnerability to splicing inhibition, such as MYC overexpression or mutations in genes encoding splicing regulatory proteins [8].

### CDKs

Inhibition of the transcriptional CDKs is emerging as a powerful tool to sensitise cancer cells that have acquired resistance to standard treatments. For instance, the highly selective and potent *CDK9* inhibitors A‐1592668 [173] and AZD4573 [174] downregulate Ser2 phosphorylation in the RNPII CTD and repress expression of MCL‐1, an important anti‐apoptotic factor that limits the efficacy of the BCL2 inhibitor Venetoclax in haematologic tumours [175]. Pre‐clinical studies showed that both *CDK9* inhibitors are well tolerated and enhance the efficacy of Venetoclax in xenograft models of acute myeloid leukaemia [173, 174]. On these bases, AZD4573 is currently being evaluated in a phase I clinical trial for haematologic malignancies (NCT03263637; Table 1). As mentioned above, these inhibitors are particularly effective in cancers that are driven by oncogenic transcription factors. For instance, inhibition of CDK9 was recently shown to repress the AR‐driven oncogenic programme in castration‐resistant prostate cancer cells *in vitro* and *in vivo* [176]. Likewise, inhibition of *CDK12* resulted in synthetic lethality in Ewing sarcomas driven by the oncogenic fusion protein EWS‐FLI1 [177], which encodes for a powerful transcription factor that reprogrammes the transcriptome of sarcoma cells [178]. It was found that *CDK12* inhibitors (THZ1 and THZ531) also impaired expression of DDR genes in Ewing sarcoma cells and sensitised them to treatment with PARPi [177]. This impact on the DNA repair proficiency of *CDK12* is likely not limited to Ewing sarcoma. Indeed, previous studies showed that *CDK12* inhibition by dinaciclib, a pan‐CDK inhibitor, sensitised to PARPi triple negative breast cancer cells that are wild type for BRCA1/2 [179]. Moreover, a recent study reported that a new selective *CDK12*/*13* inhibitor (SR‐4835) also displayed synthetic lethality in combination with DNA‐damaging agents in triple negative breast cancer cells [140]. In this regard, it is likely that the widespread dysregulation of 3'‐end processing of DDR genes caused by *CDK12*/13 inhibitors [136, 137] plays a key role in their anti‐oncogenic activity. On this basis, several clinical trials are ongoing to verify the increased sensitivity of tumours harbouring *CDK12* mutations to PARPi (reviewed in [157]). Moreover, the efficacy of THZ531 is currently being tested in an observational trial employing organoids derived from high grade serous ovarian cancer patients (NCT04555473; Table 1), to verify whether *CDK12* inhibition sensitises DDR‐proficient tumours to PARPi [180]. Unfortunately, however, clinical translation of the current *CDK12*/13 inhibitors is limited by their low efficacy *in vivo*, which is likely due to rapid efflux from the cell [181]. It is foreseen that chemical improvement of these drugs will allow their evaluation in clinical trials aimed at extending the use of PARPi also to patients that do not harbour mutations in *BRCA1/2* or other DDR genes. Moreover, similar to the effects elicited by *CDK12*/*13* inhibitors, a recent study reported that a selective *CDK7* inhibitor (SY‐1365) significantly downregulated the expression of DDR genes [182]. On the basis of its cytostatic and cytotoxic effects in many different cancer types, this inhibitor is currently being tested in a clinical trial recruiting ovarian and breast cancer patients (NCT03134638; Table 1). However, the mechanism involved in this *CDK7*‐dependent process appears different from inhibition of *CDK12*/*13*. Indeed, while repressing *CDK7* activity causes widespread changes in AS regulation, such as inhibition of *CDK12*/*13*, no significant evidence for genome‐wide premature intronic polyadenylation was observed [118]. Thus, *CDK7* and *CDK12*/*13* inhibitors likely modulate similar biological processes through different mechanisms, suggesting possible synergistic anti‐cancer effects on their combined administration.

## Future perspectives and conclusions

Pervasive amplification of transcription distinguishes many cancer cells from normal cells [183] and requires proper and efficient pre‐mRNA‐processing [184]. Therapeutic targeting of splicing is a novel area in the field of cancer therapy, with promising results related to the selective anti‐tumoral effects for cancer cells exhibiting transcriptional addiction [8, 162]. Moreover, higher sensitivity to splicing inhibitors was demonstrated in cancer cells displaying intrinsic defects in the splicing machinery (i.e. mutations in selected splicing factors or splicing‐regulatory proteins) [8, 162]. These observations support research aimed at identifying biomarkers of potential susceptibility, as well as for combined therapeutic approaches that can induce such vulnerability. In this view, the prominent role played by reversible phosphorylation of splicing regulatory proteins on essentially all the steps involved in pre‐mRNA‐processing regulation points to protein kinases as elective targets for therapy. In this regard, since kinases have been long investigated as therapeutic targets, and strategies to improve selectivity and efficacy of kinase inhibitors are well documented [185, 186], it is likely that improvement of currently promising inhibitors will be achieved in a reasonable time‐frame.

It is also worth mentioning that, in addition to the specific splicing factor kinases described in this review, other kinases may represent interesting objects of investigation and research in this field [9, 187, 188, 189, 190, 191] (Table 2). Some of these kinases, such as the mitotic kinases AURKA and NEK2, are frequently upregulated in multiple cancers and both were shown to phosphorylate and activate the splicing factor SRSF1 (Table 2), thus contributing to its oncogenic functions [190, 191]. Other kinases, such as AKT and MAPK [24, 187, 188, 192, 193, 194, 195, 196] (Table 2), are well known effectors of signaling pathways that are frequently deregulated in cancer cells. Another interesting ‘atypical’ kinase involved in pre‐mRNA‐processing is the bromodomain protein BRD4, which is better known as transcriptional regulator [196]. BRD4 displays an intrinsic kinase activity towards the RNPII CTD and was shown to modulate the transcription elongation rate [198, 199]. Moreover, BRD4 phosphorylates the oncogenic transcription factor MYC and negatively affects its stability [199]. Interestingly, BRD4 interacts with multiple splicing factors and inactivation of its expression or activity affected splicing regulation in a leukaemia cell line model [200]. Nevertheless, whether or not phosphorylation of the interacting splicing factors by BRD4 is involved in this process has not been investigated yet. This observation, together with previous findings reporting BRD4‐mediated regulation of the androgen‐insensitive AR‐V7 splice variant in prostate cancer [201], points to BRD4 as a relevant splicing modulator in cancer cells. It will be interesting to test BRD4 inhibitors, like those for the canonical splicing factor kinases, as therapeutic strategies to induce RNA‐processing vulnerability in future studies. More generally, genome‐wide elucidation of the impact of non‐canonical splicing factor kinases on the cellular transcriptome may pave the ground for their rational employment in specific tumour types.

**Table 2:** Protein kinases regulating pre‐mRNA processing.

Protein kinase	Known substrates	Regulatory effect	References
AKTs	hnRNPs, SR proteins, IWS1, CLK1, SRPK1	Modulation of RNA affinity, splicing/kinase activity and subcellular localisation	[24, 188, 192, 193, 194, 203]
AMPK (AMP‐activated protein kinase)	SRSF1	Modulation of RNA affinity	[203]
AURKA (Aurora kinase A)	SRSF1	Modulation protein stability	[189]
BRD4	RNPII	Modulation of transcriptional elongation	[198, 199]
DNA Topoisomerase I	SR proteins	Modulation of splicing activity	[204]
DYRK1A (Dual‐Specificity Tyrosine‐(Y)‐Phosphorylation Regulated Kinase 1A)	SR proteins, RNPII	Modulation of subcellular localisation and of transcriptional elongation	[206, 207, 208, 209, 210]
FASTK (Fas‐Activated Serine/Threonine Kinase)	TIA1	Modulation of splicing activity	[210]
GSK3B (Glycogen synthase kinase‐3 beta)	PSF, SRSF2, SRSF9, PSF and other splicing factors, RNPII	Modulation of RNA affinity, subcellular localisation and of transcriptional elongation	[189, 212, 213]
MAPKs (Mitogen activated protein kinases)	SAM68, SPF45, DAZAP1, SKIIP	Modulation of RNA affinity and splicing activity	[187, 195, 196, 214]
*NEK2 (NIMA–related kinase 2)*	SRSF1	Modulation splicing activity	[190]
PKA (cAMP‐Dependent Protein Kinase)	hnRNPs, SR proteins	Modulation of RNA affinity and of splicing activity	[207, 215, 216]
*PLK3 (Polo‐like kinase 3)*	RNPII	Modulation of transcriptional elongation	[216]
PRP4FB (pre‐mRNA‐processing factor 4B)	PRP6, PRP31	Modulation of spliceosome assembly	[217]
PTKs – protein tyrosine kinase	SAM68, SLM‐1, SLM‐2, RBM39, YT521‐B	Modulation of RNA affinity and of subcellular localisation	[219, 220, 221, 222]

## Conflict of interest

The authors declare no conflict of interest.

## Author contributions

CN, PB and CS conceived and wrote the manuscript.
